# Animal-Based Measurements to Assess the Welfare of Dairy Cull Cows during Pre-Slaughter

**DOI:** 10.3390/ani10101802

**Published:** 2020-10-04

**Authors:** Marlyn H. Romero, Magali Rodríguez-Palomares, Jorge Alberto Sánchez

**Affiliations:** 1 Department of Animal Health, Faculty of Agrarian and Animal Sciences, University of Caldas, Manizales 170004, Colombia; jorge.sanchez@ucaldas.edu.co; 2 Department of Ethology and Wildlife, Faculty of Veterinary Medicine, National Autonomus University of Mexico (UNAM), Mexico City 04510, Mexico; mvzpalomares@gmail.com

**Keywords:** culling, fitness for transport, slaughter, well-being, physiology

## Abstract

**Simple Summary:**

Dairy cows that are selected for culling are a vulnerable group that can experience welfare problems during pre-slaughter. This study was conducted to evaluate the animal welfare of cull dairy cows during pre-slaughter using animal-based indicators in a Colombian commercial abattoir. A total of 137 cull cows (*n* = 60 Holstein and *n* = 77 Normandy crosses) from 62 dairy farms were evaluated. Transport conditions, health indicators on arrival, human–animal interaction, physiological stress variables, carcass bruising, and the presence of pregnancy were evaluated. Results showed that 74.5% of the cows showed signs of disease, 98.5% were very thin, 84.7% showed bruising on the carcasses, and 35.7% were pregnant. The results suggest that the cows were unfit for transport before leaving the farms and that their welfare was affected by the stress generated by the challenges of pre-slaughter. There is a need to strengthen the implementation of protocols to assess the fitness of animals for transport, the awareness of farmers about the importance of animal welfare, and the training of handlers participating in pre-slaughter.

**Abstract:**

Culling is the departure of cows from the herd as a result of sale, slaughter, health, national regulations, salvage, or death. Cull cows are removed from farms with poor health, production, behavior, or other problems, and during pre-slaughter they are sometimes kept without food and water, which compromises their well-being. The objective of the present study was to evaluate the welfare state of culled dairy cows during pre-slaughter using some animal-based measurements and to identify possible associations between them. Data were recorded for 62 different dairy production farms referring to 137 cull cows (*n* = 60 Holstein and *n* = 77 Normandy crosses) slaughtered in an abattoir in Colombia (South America). In this study, we evaluated and recorded land transport conditions, the health of animals on arrival to the abattoir, human–animal interaction, stress physiological variables and the association of these variables with characteristic bruises on the carcass, the lairage time, the presence of diseases, and the stage of pregnancy. In total, 98.5% of the cows were very thin, 35.7% were pregnant, and 84.7% had bruising on the carcass. In total, 74.5% had clinical conditions; these included skin lesions (32.4%), mastitis (27.5%), lameness (21.6%), vulvar secretions (8.8%), diarrhea (6.8%), and eye carcinoma (2.9%). The total number of cull cows with bruises during pre-slaughter was associated with lot size, transport time, presence of pregnancy, body score condition, and creatine kinase levels. The results suggest that the cows were not fit for transport because their health was severely affected before they left the farms. The animal-based indicators used in this study are useful for evaluating the welfare of cull dairy cows at abattoirs.

## 1. Introduction

Culling is an important management tool in dairy herds as it affects herd economics and animal welfare [1]. Culling is the departure of cows from the herd as a result of sale, slaughter, salvage, or death [2]. Dairy cows are culled for different factors related to the presence of health problems, injury, infertility, low milk production, poor behavior, death, and old age, among others [3]. The culling of dairy or beef cattle from their respective herds is an unavoidable and necessary practice that may lead to herd improvement and increased profits, or the reduction of costs by replacing sick or non-pregnant cows [3]. The sale of cull cows represents a revenue source for dairy farms and meat originating from cull cows can represent most of the beef consumed in some countries [4,5]. Cull dairy cows are a more vulnerable category of animals than other cattle categories, because they are often made up of older animals, with poor body condition, and the presence of diseases and other conditions that produce weakness, which in turn can affect the cow’s ability to handle the stress of transportation and may increase the risk for reduced welfare [6]. It has also been noted that because of their low commercial value, and the limited incentives that the cattle logistics sector provides handlers to treat them appropriately, they are more likely to experience poor welfare during pre-slaughter [7,8].

According to Ferguson and Warner [9], the pre-slaughter phase includes the conditions and practices that apply during the period when the animals are moved or mustered on-farm to enter into the knocking box or V-restrainer at the abattoir. This stage represents a challenge for the animals because they may be exposed to a variety of stimuli including quality of handling and increased contact with handlers [10], transport conditions [11], temperature fluctuations [9], and changes in social structure [11], among other aspects. The quality of the design of the facilities and handling procedures play a key role in determining the effects of pre-slaughter on cull cow’s response to stress, as it may to reduce the handling time, staff workload, and stress in cull cows [12]. Poor handling management will impair culls cows movement during loading/unloading, leading to rougher handling and stressful experiences, and ultimately reduced welfare. Previous studies in Colombia [13] and other countries [14] have shown that carcasses from cows have more bruises than other cattle categories, which shows poor welfare during pre-slaughter stages. In the pre-slaughter stage several factors related to the presence of hematomas in the carcasses have been identified, such as: loading at the farm, stocking density in the truck, stopovers during the journey, lairage time, handling and animal collisions inside the facilities, stunning conditions, social mixing, and antagonistic encounters among the animals, among others [14,15].

In America, and other continents, cull cows are transported by land for relatively short periods of time (<5 h) and may arrive at abattoirs or livestock markets as downers or non-ambulatory [5,8]. Likewise, cull cows are kept longer in lairage, a stage in which they are grouped with other bovines from different categories (heifers, bulls, and steers), and kept without food and occasionally without water [11,14]. These factors affect their physical condition [3], increase stress and pain levels [11], increase the probability of contusions [16,17], make them difficult to control, and decreases their fitness to be considered fit for slaughter [18]. Many cows culled from dairy farms arrive at abattoirs in compromised conditions. However, few studies have been done that evaluate the adaptive costs of the animal’s reaction to stress caused by pre-slaughter management [19]. Colombian sanitary legislation in 2007 included animal welfare as a requirement to ensure the safety of beef, buffalo, and pork meat, with a farm-to-table approach [20]. Likewise, Law 1774 of 2016 recognized animals as sentient beings, highlighting the need to protect them from suffering and pain, especially that caused directly or indirectly by humans [21]. This same law establishes sanctions and fines for animal abuse. However, the level of implementation of this legislation is low and at present the state and the livestock guilds are combining their efforts for its dissemination, as well as for training and implementation. Furthermore, there is not a lot of clarity regarding the implementation of guidelines for the pre-slaughter of culled animals in Colombia based on national regulations, as well as World Organisation for Animal Health-OIE standards on animal welfare [22]. The objective of the present study was to evaluate the welfare state of cull dairy cows during pre-slaughter using some animal-based measurements and to identify possible associations between them.

## 2. Materials and Methods

The study was carried out in a commercial abattoir in the department of Caldas (central Colombia, South America) from April to June 2016. The abattoir complied with the 1500 decree [23] that created the Official System of Meat Inspection, Surveillance and Control for all meat and meat products. The animals were transported and slaughtered in compliance with national regulations applied in research and commercial slaughtering. The abattoir selected can be considered typical of the country and was selected because it was located in an area representative of Colombia’s dual-purpose dairy farming [24].

### 2.1. Ethical Note

The study was carried out under commercial conditions, and the researchers participated in the process solely as observers. Permission to conduct the study was approved by the Ethics Committee for Animal Experimentation of the University of Caldas (Act 1 07/05/2016—Activities with minimal risk). The study was designed as an observational study to evaluate clinical conditions, human–animal interactions, physiological profiles, and the evaluation of bruises.

### 2.2. Evaluation of Transport Conditions

The vehicles used (*n* = 51) and journey conditions of the cull cows were evaluated by the use of a structured survey filled in by the truck drivers (Table 1).

### 2.3. Handling of Cull Cows in the Abattoir

Data were recorded for 62 dairy production farms and included 137 cull cows (*n* = 60 Holstein and *n* = 77 Normandy crosses). The abattoir operated from Monday to Friday (09:00–18:00 h) with a slaughter capacity of 270 heads/day at a rate of 30 heads/h. At lairage, the animals had access to water ad libitum while resting. A concrete straight passageway guided animals from the lairage area to a stunning box without a head fixation system. Access to the stunning box was through a guillotine door that ejected from the side of the box. After being stunned by a non-penetrating captive bolt, the cattle were suspended by a hind leg, bled, and transferred to the production line to begin the process of removing the head, feet, skin, and viscera; the carcass was quartered.

In Colombia, the commercialization system for cull dairy cows has a high number of intermediaries. The first phase includes the participation of a purchaser who buys the animals at the farm, this person organizes a truck to pick up the cows and deliver them to the abattoir. The second phase includes an intermediary who sells the cattle to meat retailers, this transaction is carried out at the market or abattoir plant [24]. In the abattoir, they were mixed with other categories (steers, heifers, cows, and bulls) from multiple farms. Cull cows stayed up to a week in lairage because their slaughter was scheduled on different days in order to meet the weekly demand for meat from meat retailers; therefore, for the purpose of this study they were divided into two groups in accordance to the lairage time: a group with lairage times ≤ 24 h (*n* = 65) and the second for animals with lairage times > 24 h (*n* = 72). The cows were not fed after staying in the plant for more than 24 h.

### 2.4. Handling Assessment

Two postgraduate trained students (always the same people) simultaneously (one observed the cull dairy cows and the other one observed the livestock handlers), recorded the frequency of the tactile, auditory, and visual interactions used by the stockperson to move the animals [25] and the immediate cattle reactions to the handling [26]. The interaction used by the handler was observed for 30 s and the bovine behavioral response for 10 s. A bout criterion interval of 5 s was chosen to separate one bout of behavior from another bout of the same behavior; therefore, one bout of behavior was recorded during a 5 s interval [25]. The observer did not start a new measurement without the previous observation time being completed. Observations were made during unloading and handling in the corridor. The unloading time was considered as the time in which the door of the truck was opened to unload animals until the last animal entered into lairage at the abattoir. The behavioral events recorded were frequency (count) of falls (cow’s body touching the floor during handling), aggression/fight (antagonistic behavior observed among animals, like hitting with the head or horns), slips (when a cull cow lost balance temporarily), baulks (when an animal stopped suddenly and refused to walk for 10 s), reversing (when an animal moved backwards), mounting (when an animal mounted another animal), freezing (cow stops a forward movement, appears tense or fearful, it’s ears forward, with full attention focused on an object), and vocalization (bellow, moo).

The frequencies of interactions were recorded as tactile, auditory and visual. Tactile interactions of humans included pushing, hitting (when the handler hits with the hand or an object), prodding (when the handler prods with an object such as a wooden stick) and electric shocks. Additionally, tail twisting and prods to sensitive parts of the animal such as the eyes, ears, nose, anus or testicles were recorded as negative human interactions. Auditory interactions included talking, whistling and the use of artificial noises, such as banging of pen fittings. Waving was the only visual interaction recorded [25].

### 2.5. Clinical Conditions Assessment

Animals were assessed individually by a veterinary during lairage at the abattoir. The assessment included general condition, body conditions scoring (BCS), and quality defects that could affect the cow’s suitability for transport and slaughter. BCS was scored using a scale from 1 to 5 with quarter point increments, where 1 was a very thin cow and 5 a very obese cow [18]. Locomotion was scored using a scale from 1 to 5, where 1 was a cow that walked normally and 5 a severely lame cow [27]. Cows were scored on a solid concrete floor while walking from the unloading area to the lairage pen. Cows included in the dataset were scored as lame with a locomotion score of at least 3, in order to code this variable in two categories: presence (score ≥ 3) or absence of lameness (score ≤ 2). The evaluated clinical conditions were recorded as a dichotomous variable that was coded as 1 = signs of disease/weakness and 0 = absence of signs of disease/weakness, including signs of pneumonia, secretions (nasal, ocular, or vulvar), gastrointestinal conditions (severe diarrhea), and eye carcinoma. The presence or absence of pregnancy and mastitis were determined in the post-mortem inspection. The presence of pregnancy and mastitis was detected in the viscera inspection room; in the first case by visual inspection and incision of the reproductive tract. The presence of mastitis was evaluated by visual inspection and palpation of the udder, and incision of the supra-mammary lymph nodes.

### 2.6. Physiological Measurements

For lab analysis, blood samples were collected during bleeding (two 10 mL tubes per animal-BD Vacutainer, Franklin Lakes, N.J., with and without anticoagulant, EDTA). Samples were kept on ice during sampling (up to 2 h) and then taken to the laboratory for routine hematological measurements. Packed cell volume (PCV) values were obtained using the micro hematocrit technique. Serum cortisol concentrations (μg/dL) were measured in duplicate using a radioimmunoassay (RIA) (Clinical Assays Gamma Coat Cortisol 125I RIA Kit, DiaSorin, New York, NY, USA), the coefficient of variation inter-assay was 9.31%. The concentrations of glucose (mmol/L), creatine kinase (CK) (U/L), and creatinine (mmol/L), were determined using a Bio-system kit (Biosystems^®^, Barcelona, Spain) and spectrophotometer BTS-330 (Biosystems^®^, Barcelona, Spain). The evaluation of the unsterilized fatty acid (NEFA, mmol/L) levels, β-hydroxybutyrate (BHBA mmol/L), and lactate (mmol/L) concentration was performed with Randox kits (Randox Laboratories Limited^®^, Crumlin, United Kingdom) and a spectrophotometer BTS-330 (Biosystems^®^, Barcelona, Spain).

### 2.7. Bruising Assessment

The protocol for the post-mortem evaluation was based on the Colombian bruising carcass-grading standard described in [15]. This observation was done after the de-hiding point and before the carcass-splitting point on the slaughter line, allowing the entire carcass—hanging by both hind legs—to be easily observed. In the case of each carcass, the presence of bruises (positive or negative) was recorded first. If bruises were present, the number of bruises per carcass and the number of bruises per anatomical site were assessed. Next, each bruise present on the carcass was evaluated by registering its anatomical site, size, severity, and shape. The original Australian Carcass Bruising Scoring System, ACBSS score sheet for half a carcass was extended to allow a complete recording of bruises for the entire carcass. The anatomical site of the bruise was recorded according to the ACBSS, and the carcass was divided into seven areas: anatomical location 1 = lateral side of the hind leg, 2 = abdominal wall, 3 = thoracic wall, 4 = front leg, 5 = loin, 6 = Tuber isquiadicum and its muscular insertions, 7 = Tuber coxae and its muscular insertions, and 8 = multiple areas affected. The severity of the bruise was rated by the observer: grade 1: only subcutaneous tissue affected; grade 2: as grade 1, but with muscle tissue affected; grade 3: as grades 1 and 2, but with the presence of broken bones. The following shapes were differentiated; circular: a bruise shaped like or nearly like a circle; linear: a noncircular bruise with one dimension (length) longer than the other (width); tramline: two parallel linear bruises separated by a paler undamaged area; mottled: the bruised area appears spotted or blotched; and irregular: a bruise without clear dimensions and with uneven edges.

### 2.8. Data Handling and Statistical Analyses

The software Stata Version 13.0 (College Station, Texas, EU) was used for all the statistical analyses. First, a normality test of the evaluated variables was carried out and the variables with non-normal distribution were transformed by means of the natural logarithm; these values were used for later statistical analysis. A multilevel analysis was carried out to establish the effect of the cows’ farm of origin variable, in the models for the analysis of continuous and categorical dependent variables, where it was verified that it had a minimum effect. Multiple linear regressions were used in order to test the relationship between physiological stress indicators (dependent variables) and independent variables (transport time, stocking density, lairage time, flooring space at lairage, delay time, live weight at slaughter, pregnancy, disease presence, and lot size); selection of variables was done using the forward method and models’ fit was tested by means of the adjusted R^2^. Pearson correlation (r) was used to measure the degree of relationship between the interactions of handlers (per min) and the number of different cattle behaviors (per min). A one sample t-test was used in order to test the mean difference between lairage times (≤24 h vs. >24 h), the behavioral and human–animal interactions recorded during unloading and handling in the corridor, presence of pregnancy, and presence of diseases. A negative binomial regression was used in order to test the relationship between number of bruises and independent variables (transport time, stocking density, lairage time, flooring space at lairage, delay time, live weight at slaughter, pregnancy, disease presence, and lot size), selection of variables were done using the forward method (∞ = 0.05) and models’ fit was tested by the χ^2^ Pearson test. A relative risk (RR) that is greater (smaller) than 1 indicates that the variable is more (less) likely to be present in a specific category of the predictor variable compared to the reference category. A probability level of *p* < 0.05 was chosen as the limit for statistical significance in all tests.

## 3. Results

The least square means of the pre-slaughter management practices, such as stocking density, transport time, and live weight at slaughter, were significantly different between the two groups of cows according to lairage times at the abattoir (Table 2). The group B cull cows (lairage time > 24 h) were kept in the abattoir for an average of 3.5 days.

### 3.1. Handling Assessment

During loading the animals had higher frequencies of slips, falls, reversing, vocalization, and baulks compared to driving in the abattoir corridor, and these differences were statistically significant (*p* < 0.05) (Table 3). Auditory interactions (whistling and talking) were more frequent during unloading (0.52 ±0.05, 0.81 ± 0.4), while tactile interactions (electric shocks, 2.95 ± 0.04)) and artificial noises (0.16 ± 0.01) predominated during driving in the abattoir corridor (2.21 ± 0.01). The artificial noises and hitting generated reverse reactions which were significantly correlated (*p* < 0.05). During the unloading process handlers stimulated the driving of the animals by twisting their tails, thereby generating a high proportion of baulks, reactions which were significantly correlated (57.14%, *p* < 0.05). It is noteworthy to mention that aggressive behavior of the cows towards the handlers was at a low frequency. Likewise, the most frequent behavioral response of the cull cows during handling was freezing (1.51 ± 0.05 and 3.81 ± 0.05) (Table 4).

### 3.2. Condition of Cull Cows

Most cows had a BCS of 1 (*n* = 71; 51.8%), a slightly smaller proportion had a BCS of 2 (*n* = 64; 46.7%), and only 1.5% (*n* = 2) were scored with a BCS of 3. In total, 35.7% (*n* = 49) of the cows were pregnant and 74.5% (*n* = 102) had quality defects; these included skin lesions (32.4%, *n* = 33), mastitis (27.5%, *n* = 28), lameness (21.6%, *n* = 22), vulvar secretions (8.8%, *n* = 9), diarrhea (6.8%, *n* = 9), and eye carcinoma (2.9%, *n* = 3).

### 3.3. Physiological Measurements

The means of measuring the physiological welfare indicators of cull cows at abattoirs are presented in Table 5. The basal level of cortisol, glucose, protein, albumin, CK, lactate, NEFA, hemoglobin, and the ratio N/L were higher in cull cows with lairage times ≥24 h (*p* ≤ 0.05). Results from the multiple linear regressions for transformed blood values of CK, hemoglobin, lactate, creatinine, and NEFA showed significant (*p* ≤ 0.05) relationships among transport time, lairage time, unloading time, body condition, stock density, lot size, live weight, presence of disease, and pregnancy (R^2^ ranged from 0.115 to 0.321) (Table 6).

### 3.4. Bruising

During post-mortem evaluation, a high proportion of bruised carcasses (84.7%) as well as multiple bruises per carcass (2.1 ± 1.2) were found. The bruises were most frequently found on the tuber coxae (30.5%) and the tuber isquiadicum (27.2%) sites of the carcasses. Of all bruises, 90.6% were grade 1, and most had an irregular shape (90.8%) and showed a diameter between 2 and 8 cm (62.8%) (Table 7). Significant differences (*p* < 0.05) were observed in the number of bruises/animal, anatomical areas, size and shape of bruises in cows that remained in longer lairage time at the abattoir. The total number of bruises of cull cows during pre-slaughter were associated with lot size, transport time, presence of pregnancy and body condition score (BSC) (Table 8).

## 4. Discussion

### 4.1. Handling Assessment

Generally, behavioral observations are valuable for the monitoring of dairy cows’ health and welfare. In the present study, like other studies carried out in Colombia [13,28], France [29], and Sweden [30], compared to unloading, the moving of animals through the abattoirs’ corridors was the most difficult part for the handlers and produced the most reactions from the animals (slips/falls, reversing, vocalizations, and baulks). These differences can be put down to the characteristics of the installation, the animals fear, the cows flight reactions stimulated by human interaction, a new environment, light, and noise, among other reasons. In this study, handling difficulty in the corridors is manifested by the use of electric goads and artificial sounds to keep them moving. Electric goads are very stressful for cattle and produce a dramatic change in their behavior and an increase in animal vocalization [30]. This study shows that the use of electrical shocks produced negative reactions in the animals that were difficult to reverse like slips, jumps, vocalization, and falls. This suggests that handlers need to be trained to better handling natural animal behavior, have a rigorous selection process for personnel, give incentives to those personnel that handle the animals correctly, implement cognitive/behavioral interventions (like handling skills training) and limit the use of electric goads [31]. Colombian legislation prohibits the use of blunt, sharp, or electric instruments for handling cattle on farms Resolution 2341 of 2007, but does not specify these same conditions in abattoirs [20], which is why electric goads are used for handling cattle. However, this practice is in decline because the producer associations have recognized the importance of animal welfare as an added value to the quality of food of animal origin [24]. As regards interactions like whistling and speaking, various studies have concluded that they have a neutral effect on the cattle behavior [29,30]. We suggest that handling the cattle in this way can have a calming effect and alert the cattle that the handler is approaching, especially as these are common methods employed in Colombia; as has been shown in livestock abattoirs and auctions [32].

Animals differ in their reaction of stressful situations and consequently in their stress status. These variations may be explained by interindividual differences in emotional reactivity, defined as the animal’s tendency to show more or less pronounced reactions to different fear-inducing situations [19]. Experimental studies have suggested that the reaction of cows in pre-slaughter can be influenced by being in an unfamiliar environment, social separation, and the capacity to perceive stress among animals from the same group [19]. It is possible that the low aggressive reactivity of the cows in this study to the handling of the personnel is associated with a high level of human acceptance due to dairy systems requiring close contact every day [31]. In the same way cull cows displayed a predominant behavior of freezing in the face of negative human interactions; this is considered a fear response from cull dairy cows, making them more fearful and difficult to handle [33], which is the reason why we propose that aggressive handling is unnecessary and that the attitude of personnel towards the animals can be modified to favor a more positive work environment and job satisfaction; this can be done by reinforcing good handling practices and encouraging positive attitudes among the personnel towards the animals.

### 4.2. Clinical Condition Assessment

Body condition scoring is a subjective technique that assesses the proportion of fat and muscle in the live cow and is widely accepted as an indicator of past nutritional status. Colombian legislation has established sanitary and safety requirements for the primary production of bovine cattle destined to be slaughtered for human consumption. The guidelines include information about primary production and transportation [34] as well as the requirements that must be met for animals to be considered for transportation, slaughter, and consumption [35]. This regulation prohibits the transport of animals that are sick, weak, or in an advanced state of pregnancy. However, this research found a high proportion of animals with a body condition of one or two, categories that reflect emaciation, malnutrition, and poor health [11]. These findings agree with other investigative reports in North America [36], Canada [6,16], Chile [8], and Denmark [37]. Low BSC values may be associated with the presence of wasting diseases, lack of food and water supply during pre-slaughter, regrouping, and other stressors that may have initiated the mobilization of the body’s energy reserves [6]. These results indicate that the clinical condition of cull cows at the end of their productive life has not received much attention, although it is highly relevant information to improve the welfare condition of these animal, as well as fitness for transport and product quality [8].

The selecting process of cull cows by the producers is complicated, it involves both criteria linked to individual production (milk production level, stage of lactation, reproductive status, age, genetic value, and health problems) and herd level factors (herd behavior or group cohesion, production needs, availability of replacement animals, price of milk and culled, cows, etc.) [38]. Regarding health problems, to guarantee food safety some studies carried out in Canada have shown that this aspect is one of the main interests of producers and consumers [38,39]. However, various studies have shown that a high proportion of cull cows enter abattoirs in emaciated conditions and showing signs of sickness like eye carcinoma, lameness [40], udder defects [36], and wounds [37]; results consistent with those obtained in the present investigation. This aspect may be associated with the producers’ perception of their animals. Several researchers have suggested that producers who see their animals as mere tools for production take decisions based on economic interests, and animal welfare is seen as a secondary aspect [41]. In contrast, dairy cattle producers who aim to expand their business and have higher levels occupational well-being see the implementation of animal welfare as a production strategy [42].

One of the primary reasons for culling in the investigations is usually poor udder health/mastitis and lameness [5], results that agree with those obtained in this study. Dairy cow lameness and mastitis are the focus of much scientific attention and are widely recognized as painful conditions leading to reduced animal welfare [27]. In our study, 27.5% of cows had mastitis and 21.6% had lameness. During mastitis, the concomitant inflammation of the udder, increased intra-mammary pressure, and increased external pressure (e.g., from an adjacent limb on a swollen udder) are believed to induce pain [43]. However, some authors consider that mild clinical mastitis problems should not be considered as a condition that establishes the inability of a cow to be transported [5]. This is because minor injuries may not cause enough pain to observe marked changes in animal behavior [44]. In contrast, the authors of [12] suggest that mastitis is one of the main conditions to be considered when thinking about unsuitability for transport, because even relatively mild cases of mastitis cause pain. This aspect would require additional studies and new validating protocols to comprehensively assess the presence or absence of pain associated with mastitis, and how the impact of these clinical findings would be used to establish the fitness of animals for transportation.

Likewise, lameness in dairy cows is second only to mastitis in terms of its detrimental effect on herd productivity, because lameness decreases milk yield, fertility, and dairy cow survival, in line with the severity of the lesions and the time of diagnosis [45]. As well as productivity losses, lameness severely compromises the welfare of affected animals and is probably the single most common cause of distress in dairy cattle [37]. The cows in the study were selected by the farmer for culling following normal procedures and as such should be representative of cull dairy cows in the study area. Taking this into account, we can assume that in Colombia and in other countries, the transport of cows with lameness and mastitis is a common event and, therefore, studies are required to evaluate the impact of this clinical condition as a risk factor of “additional suffering” for cull cows during transport and slaughter.

Under normal circumstances, pregnant females that have exceeded 90% of the gestation period should not be transported or slaughtered according to The World Organisation for animal Health (OIE) guidelines [22] and Colombian law [34], in the latter case, legislation does not discriminate the gestation time. During this study, a large proportion of cull cows were found to be pregnant (35%), and although the time of gestation was not established, the analysis is relevant from the point of view of animal welfare during transport and slaughter; taking into account that other studies have established that pregnancy is a protective factor, which reduces the probability of culling dairy cows [46].

### 4.3. Physiological Assessment

Many cull dairy cows enter the marketing system and travel to widely dispersed and specialized abattoirs, and they may experience multiple handling events (e.g., loading, unloading, transport, and mixing), change of ownership among dealers, and feed and water deprivation during the pre-slaughter process; these events produce stress and can affect their physiological profile [16]. In this study, regardless of the lairage time, pregnancy, or clinical condition, all the animals evaluated had higher cortisol and glucose levels than the basal levels of the specie (0.61 ± 0.07 µg/dL and 2.5–4.2 mmol/L, respectively); values that are suggestive of high stress and in accordance with results presented by the authors of [47] in Zebu steers in Colombia. The management conditions of the cows evaluated in this study, could also influence the N/L ratio, as it has been shown that high levels of corticosteroids released during stress produce neutrophilia and lymphopenia [47]. Short-term stress or physical exercise increases circulating adrenaline levels, degrading muscle glycogen that is used for gluconeogenesis in the liver and an increase in lactate levels. Therefore, glycogen can be used to assess animal welfare in stressful situations such as handling and poor quality facilities which increase the energetic cost for animals [43]. In this study, lactate concentration was significantly higher in all culls, suggesting that multifactorial environmental causes may affect welfare [28].

Circulating concentrations of NEFA and BHBA measure aspects of the success in adaptation to a state of negative energy balance. The concentration of serum NEFA reflects the magnitude of fat mobilization, whereas the concentration of BHBA reflects the completeness of oxidation of fat in the liver [48]. In this study, increased levels of glucose, protein, and NEFA may be associated with a lack of food supply during transport and lairage as well as stress, which is why there is an energy deficit that generates the activation of metabolic pathways such as lipolysis, β-oxidation, and ketogenesis [49]. The lack of differences between cull cows indicate that food deprivation for five days was counteracted by BHBA provided by the rumen, where multiple days are required for cattle to reach a physiological fasting state [50]. These same results have been observed in lactating cows with short-term nutrient restriction, which did not alter BHBA concentration for cows in mid and late lactation [51].

As an indicator of dehydration, all the cull cows in the study had increased hematocrit levels. Despite the abattoir’s pens having drinking fountains, the animals were regrouped in large batches; this is something that could hinder access to drinking water as well as the novelty of the facilities and equipment, the presence of dominant animals and other factors. The increase in hematocrit is attributed to the splenic contraction as a consequence of the acute stress caused by loading, transport and unloading; and in part to the decrease in plasma volume. Splenic contraction is one of the effects of the release of catecholamine by the sympathetic pathway of the autonomic nervous system [50].

Plasma lactate is an indicator of acute stress related to handling conditions, especially physical exercise, agitation, and muscular damage [50]. On the other hand, the CK activity is a sensible indicator of muscular damage, increased physical activity, and muscular fatigue [52]. In our study, CK and lactate activity drastically increased in all the cows, surpassing the average reference values [53]. The high values of both could have been related to cattle handling during truck unloading, transportation and lairage. These activities may have increased membrane permeability or harmed muscle cells and subsequent enzymatic excretion [54]. The evaluation of bruises on the carcasses showed muscle damage; an aspect to be discussed later.

Lairage is a common commercial practice that allows animal resting after transport. Animals must have access to water, and in Colombia animals spending >24 h in lairage must be fed [28]. If conditions are good, lairage may allow the replenishment of muscle glycogen concentrations and mitigate dehydration and carcass weight loss [55]. The multiple linear regression analysis showed that plasma hemoglobin (R^2^ = 0.326), lactate (R^2^ = 0.204), creatinine (R^2^ = 0.105), and NEFA (R^2^ = 0.191) levels depended on the lairage time and other variables. In the lairage, cull cows are expected to recover the energy lost during transportation. However, the rate which energy is gained depends upon the amount of stress from transportation and the conditions of the lairage at the abattoir [56]. Although a significant relationship was observed, the R^2^ value was low, indicating that the effects were small.

### 4.4. Bruise Assessment

Bruises can be a source of pain and fear for animals because a bruise could make a cow fearful of suffering additional pain from an event over which it may have little control and predictability [14]. In Colombia, there have been several studies that assess the presence of bruises on carcasses during cattle and porcine slaughter [13]. The overall bruising prevalence observed here is numerically higher than that found by the other studies in cull cows in the Great Lakes region of the United States (54.1%) [3], and in Chile (71.5%) [14]. Diversely, bruises were chiefly located in the ischial/coccygeal tuberosities and agreed with results of other studies in Colombia [15]. These lesions may have been caused by loading and unloading procedures, transport, equipment problems, collision of animals with structures, and to group mixing [13], and may also be due to the fact that this anatomical area has less tissue density and fat cover [57]. In this study, a higher proportion of bruises was observed in the females that stayed longer in lairage, perhaps as a result of more severe antagonistic encounters between the animals, the mix of different cattle breeds, staff handling difficulties and the use of negative interactions, as well as high stocking densities in the pens, among other aspects [14].

Short journeys are a characteristic of Colombian export abattoirs, because they are located in areas with great livestock production, unlike the abattoirs for the national market that receive animals that have been transported for more than 10 h [15]. In our study, the abattoir was located in a dairy region, which is why cull cows presented short transport times. However, the transport time was a factor that affected the total number of bruises. On the other hand, lot size depends on load density, and load density is one of the factors that should be considered in the evaluation of animal welfare during transportation; this is because inadequate densities due to excess or lack of space for the animals may result in reduced carcass quality and the presence of bruises [58].

Lot size during transport can increase the probability of animal–animal, human–animal, and animal–facility interactions [14]. Lower fat coverage and extreme thinness are factors that also increase this risk [57]. It is possible that pregnant cows, having greater volume and weight, present a greater probability of slipping, falling, and colliding against the infrastructure, an aspect that would explain their greater risk of presenting bruises.

Two limitations have been identified in this study: (1) measurement biases, related to the participation of two observers in the recording of information, and (2) selection biases, because the study is carried out in a single abattoir and the difficulty in generalizing the results. These biases were controlled by the authors through the training of the observers in the evaluation of human–livestock interaction and the evaluation of hematomas in the pilot test. The previous conditioning period of one month was used to gain the confidence of the handlers. In relation to the representativeness of the abattoir evaluated, the authors, taking into consideration research on bovine pre-slaughter and their experience in the region, consider that this plant is representative of Colombian commercial abattoirs. However, the results cannot be generalized to all abattoirs in the country, due to differences related to infrastructure, management, organizational characteristics, handlers’ levels of training and education in animal welfare, and clinical conditions of the animals, among other aspects.

## 5. Conclusions

The management of cull cows during pre-slaughter represents a serious problem for animal welfare because only 1.5% presented an acceptable body condition, and a high proportion of animals (74.5%) were sick and pregnant (35.7%), indicating that their fitness was compromised before transport. The results of this study show a need for clear and specific guidelines to assess fitness for transport of cull dairy cows. This lack of guidelines and producer knowhow to select cull cows for transport in an appropriate manner has negative consequences on the welfare of the animal, especially because the animals in cachectic and weak conditions were physiologically challenged and subjected to long lairage hours an aspect that affected their general condition. Likewise, it shows that although there are OIE regulations and guidelines to guarantee appropriate transport and slaughter, in practice producer hardly apply them. Long lairage times, the mixture of animals of different breeds, and the lack of the food and water supply, suggest the need to raise awareness in the trade about proper cow management and the implementation of animal welfare policies in the pre-slaughter logistics chain.

## Figures and Tables

**Table 1:** Variables registered on the transport conditions, unloading and lairage of the cull dairy cows.

Variable	Description
Farm (name)	The cows’ farm of origin
Transport time (h)	Transport duration
Stocking density (kg/m^2^)	Space required per animal during transport
Lot size (number)	Number of cull dairy cows in a truck
Delaty time (min)	Time to wait in line to unload
Live weight at slaughter (kg)	Cows’ live weight at slaughter
Unloading time (min)	The time in which the door of the truck was opened to unload animals until the last animal entered into the lairage at the abattoir
Lairage time (h)	Lairage duration

**Table 2:** Least square means (±SE) of the pre-slaughter management practices at two types of lairage time at the abattoir.

Variables	Lairage Time		
	≤24 h (*n* = 65)	>24 h (*n* = 72)	*p*
Transport time (h)	3.1 ± 0.40	2.0 ± 0.25	0.02
Stocking density (kg/m^2^)	311.4 ± 16.5	372.1 ± 14.5	0.01
Lairage time (h)	21.5 ± 1.4	80.0 ± 3.04	0.01
Delay time (min)	5.4 ± 0.27	5.7 ± 0.31	0.3
Unloading time (min)	5.1 ± 0.82	6.8 ± 1.06	0.08
Live weight at slaughter (kg)	444.9 ± 10.1	411.4 ± 7.5	0.03

**Table 3:** Least square means (±SE) of interactions of stockperson (per minute) and individual behavioral events of dairy cull cows (per minute) observed during unloading and handling in the corridor at the abattoir.

Variables	Unloading	Handling in the Corridor	*p*
**Behavioral events**			
Slips	0.22 ± 0.05	0.45 ± 0.15	0.03
Falls	0.28 ± 0.05	1.23 ± 0.5	0.01
Reversing	0.47 ± 0.05	1.80 ± 0.04	0.02
Vocalization	0.10 ± 0.05	0.63 ± 0.01	0.01
Baulks	1.81 ± 0.05	0.67 ± 0.06	0.02
Mount	0.02 ± 0.00	0.03 ± 0.00	0.3
Aggression/fight	0.04 ± 0.01	0.02 ± 0.00	0.08
Freezing	1.51 ± 0.05	3.81 ± 0.05	0.01
**Interactions**			
Whistling	1.15 ± 0.4	0.10 ± 0.05	0.01
Talking	0.52 ± 0.05	0.20 ± 0.03	0.04
Hitting	0.81 ± 0.4	0.09 ± 0.01	0.01
Prodding	0.46 ± 0.07	-	0.04
Pushing	0.06 ± 0.01	-	0.03
Tail twisting	0.02 ± 0.0	-	0.04
Artificial noises	0.16 ± 0.01	2.21 ± 0.01	0.03
Electric shocks	-	2.95 ± 0.04	0.01

**Table 4:** Pearson correlation coefficients (r) between the interactions of the handlers and behavioral events of dairy cull cows during handling at abattoir.

Variables	Freezing	Falls	Slips	Jumps	Reversing	Aggression	Baulks	Mounting	Vocalization
Unloading									
Whistling	0.55 *	0.20	−0.04	2.60	0.12	0.25	−0.03	0.50	0.87
Talking	0.33	0.16	0.08	−0.20	−0.01	0.63	0.15	0.31	0
Hitting	0.21	0.39	0.01	0.67	0.57 *	0.33	0.69 *	0.33	0.67
Prodding	0.02	0.12	0.11	-	0.09	0.01	−0.01	0	0.13
Pushing	0.25	0	0.05	0.04	0	0	0.32	0	0
Artificial noises	0.65 *	0.08	0	0	0.25	0	−0.05	0	−0.20
Waving	0	0	0	0.05	0	0	0	0	0
**Handling in the Corridor**									
Tail twisting	0.29	0	0	0	0	0	0.63 *	0	0
Whistling	0.58 *	0	0	0.02	0	0	0.06	0.51	0
Talking	0.35	−017	0.10	−0.06	0	0	0.23	0	0
Hitting	0.33	0.40	0.02	0	0.60 *	0	0	0	0
Artificial noises	0.58 *	0.04	0.28	0.06	0.63 *	0.16	−0.01	−0.05	5.61
Electric shocks	−0.03	0.18	0.55 *	0.01	0.06	0.62	−0.14	0.26	0.51 *

* Significant correlation (r > 0.5; *p* < 0.05).

**Table 5:** Least square means of the physiological welfare indicators of cull cows at two different times of lairage, pregnancy, and disease presence.

Variables	Lairage Time		Pregnancy		Disease Presence	
	≤24 h	>24 h	No	Yes	No	Yes
Cortisol (µg/dL)	1.23 ± 0.15	1.37 ± 0.17	1.29 ± 0.16	1.31 ± 0.15	1.71 ± 0.27 ^a^	1.16 ± 0.12 ^b^
Glucose (mmol/L)	5.49 ± 0.14	5.78 ± 0.19	5.66 ± 0.15	5.62 ± 0.19	6.11 ± 0.16 ^a^	5.48 ± 0.15 ^b^
Creatinine (mmol/L)	143.7 ± 3.55 ^a^	166.5 ± 4.53 ^b^	148.7 ± 3.77 ^a^	167.9 ± 4.8 ^b^	157.6 ± 7.4	155.0 ± 3.2
Urea (mmol/L)	8.03 ± 0.27	8.1 ± 0.31	7.81 ± 0.26	8.6 ± 0.32	7.74 ± 0.32	8.23 ± 0.25
Protein (g/L)	8.03 ± 0.08	8.12 ± 0.09	8.09 ± 0.08	8.07 ± 0.08	8.09 ± 0.08	8.07 ± 0.08
Albumin (g/L)	3.9 ± 0,06 ^a^	4,2 ± 0,07 ^b^	3.96 ± 0.06 ^a^	4.2 ± 0.08 ^b^	4.41 ± 0.09 ^a^	3.93 ± 0.05 ^b^
CK (U/L)	496.5 ± 82.5 ^a^	315,9 ± 38,6 ^b^	375.0 ± 53.1	447.8 ± 80.2	307.6±36,4	433.8±58.3
Lactate (mmol/L)	4.42 ± 0.18 ^a^	5.86 ± 0.26 ^b^	4.91 ± 0.20 ^a^	5.64 ± 0.30 ^b^	6.01 ± 0.39 ^a^	4.89 ± 0.18 ^b^
NEFA (mmol/L)	0.56 ± 0.04 ^a^	0.71 ± 0.52 ^b^	0.59 ± 0.04	0.71 ± 0.06	0.68 ± 0.08 ^a^	0.62 ± 0.03 ^b^
BHBA (mmol/L)	0.34 ± 0.26 ^a^	0.47 ± 0.8 ^b^	0.38 ± 0.02	0.46 ± 0.03	0.37 ± 0.03	0.42 ± 0.02
Haematocrit (%)	44.3 ± 1.1 ^a^	50.3 ± 0.75 ^b^	46.5 ± 0.88 ^a^	49.3 ± 1.2 ^b^	48.8 ± 1.33	47.1 ± 0.85
Hemoglobin (g/dL)	14.6 ± 0.43 ^a^	16.7 ± 0.25 ^b^	15.4 ± 0.33	16.4 ± 0.40	16.2 ± 0.44	15.6 ± 0.31
Ratio (N/L)	2.57 ± 1.10	1.60 ± 0.08	1.55 ± 0.07	2.94 ± 1.42	1.47 ± 0.13	1.25 ± 0.69

^a^, ^b^: different lower case superscripts in the same row indicate differences statistically significant (*p* ≤ 0.05). BHBA: ß-hydroxidebutyrate, NEFA: Non-esterified fatty acid, CK: creatinine kinase, ratio (N/L): neutrophil/lymphocyte ratio.

**Table 6:** Results from multiple linear regression models of the relationship between transformed blood values of creatine kinase (CK), hemoglobin, lactate, creatinine, and non-esterified fatty acid (NEFA) with the independent variables: Y = β_0_ + β_1_Z_i_ + ε_i_, where Y is interactions (per min), β_0_ (intercept), β_1_ (slope), Z_i_ (predictive variable), and ε_i_ (error).

Variables	CK			Hemoglobin			Lactate			Creatinine			NEFA		
	R^2^ = 0.115			R^2^ = 0.321			R^2^ = 0.187			R^2^ = 0.116			R^2^ = 0.174		
	ß	Error	p	ß	Error	p	ß	Error	p	ß	Error	p	ß	Error	p
Transport time													0.08	0.021	0.00
Lairage time	−0.003	0.002	0.084	0.001	0.0005	0.01	0.003	0.0008	0.00	0.001	0.0006	0.01	0.005	0.001	0.001
Unloading time				−0.000	0.00004	0.00							−0.000	0.0001	0.014
Body condition				0.136	0.040	0.00									
Pregnancy							0.118	0.064	0.07	0.119	0.04	0.017			
Stock density										−0.00	0.0001	0.061			
Lot size	0.061	0.017	0.001	0.011	0.005	0.021				0.013	0.005	0.025			
Live weight	−0.001	0.0009	0.063	0.0000	0.000	0.004									
Disease							−0.16	0.070	0.018						

The missing variables indicate that they were not predictive in the regression models.

**Table 7:** Bruising characteristics according to the lairage time type (≤24 h vs. >24 h) of cull cows at abattoir (*n* = 433 bruises; *n* = 195 vs. *n* = 237).

Variable	Category	Total N (%)	Lairage Time	
			≤24 h Bruises/Animal	>24 h Bruises/Animal
Anatomical areas	Lateral side of the hind leg	25 (5.8)	0.14	0.14
	Abdominal wall	3 (0.7)	0.03	0.01
	Thoracic wall	36 (8.3)	0.37 ^a^	0.20 ^b^
	Front leg	36 (8.3)	0.35 ^a^	0.20 ^b^
	Loin	83 (19.2)	0.67	0.55
	Tuber isquiadicum	118 (27.2)	1.07 ^a^	0.72 ^b^
	Tuber coxae	132 (30.5)	0.77 ^a^	1.0 ^b^
Depth and severity of bruise	Grade 1	401 (92.6)	3.0	2.58
	Grade 2	30 (6.9)	0.22	0.19
	Grade 3	2 (0.5)	0.01	0.02
Size of bruise	Small (<8 cm)	272 (62.8)	1.77 ^a^	1.88 ^b^
	Medium (<16 cm)	127 (29.3)	1.18 ^a^	0.72 ^b^
	Large (>16 cm)	34 (7.9)	0.26	0.27
Shape of bruise	Irregular	393 (90.8)	3.0	2.6
	Circular	5 (1.2)	0.03	0.03
	Linear	8 (1.8)	0.07 ^a^	0.03 ^b^
	Mottled	7 (1.6)	0.11	0.11
	Tramline	20 (4.6)	0.01 ^a^	0.05 ^b^

^a^, ^b^: different lower case superscripts in the same row indicate differences between lairage time type (*p* ≤ 0.05).

**Table 8:** Effect of the independent variables on total number of bruises of cull cows during pre-slaughter. The model was $P\left(k\;events\;in\;interval\right)=e^{-\lambda }\frac{\lambda^{k\;}}{k!}\;,$ where *λ* is the average number of events per interval, *e* is the Euler´s number, k takes values 0, 1, 2, ..., and *k*! = *k* × (*k*−1) × (*k*−2) × … × 2 × 1 is the factorial of *k*.

Variables	Coefficient	Standard Error	Confidence Interval (95%)	RR *	*p* Value
Lot size	0.049	0.014	0.020–0.078	1.05	0.001
Transport time	−0.003	0.112	−0.006–0.0007	1.0	0.014
Pregnancy	0.203	0.108	0.001–0.536	1.33	0.025
Body condition	0.254	0.121	0.154–0.493	1.29	0.037

* *RR*= Relative risk.
